# Speech recognition index of workers with tinnitus exposed to environmental or occupational noise: a comparative study

**DOI:** 10.1186/1745-6673-7-26

**Published:** 2012-12-22

**Authors:** Márcia Soalheiro, Lucelaine Rocha, Diane Francis do Vale, Viviane Fontes, Daniel Valente, Liliane Reis Teixeira

**Affiliations:** 1 Centro de Estudos da Saúde do Trabalhador e Ecologia Humana, Escola Nacional de Saúde Pública, Fundação Oswaldo Cruz, Sala 55 Rua Leopoldo Bulhões, 1480, Manguinhos, Rio de Janeiro, RJ, CEP: 21040-360, Brasil; 2 Municipal Secretariat of Health and Civil Defense, Rio de Janeiro, Brazil

**Keywords:** Tinnitus, Noise, Workers’ health, Hearing health

## Abstract

**Introduction:**

Tinnitus is considered the third worst symptom affecting humans. The aim of this article is to assess complaints by workers with tinnitus exposed to environmental and occupational noise.

**Methodology:**

495 workers went through an epidemiological survey at the Audiology Department of the Center for Studies on Workers’ Health and Human Ecology, from 2003 to 2007. The workers underwent tonal and vocal audiometry, preceded by a clinical and occupational history questionnaire. Two-factor ANOVA and Tukey were the statistical tests used. All the analysis set statistical significance at α=5%.

**Findings:**

There was a higher prevalence of occupational tinnitus (73.7%), a predominance of female domestic workers (65.4%) in cases of environmental exposure, and predominance of male construction workers (71.5%) for occupational exposure. There was a significant difference in workers with hearing loss, who showed a mean speech recognition index (SRI) of 85%, as compared to healthy workers with a mean SRI greater than 93.5%. Signs and symptoms, speech perception, and interference in sound localization with the type of noise exposure (environmental versus occupational) comparisons found no significant differences.

**Conclusion:**

Studied group’s high prevalence of tinnitus, major difficulties in speech recognition with hearing loss and the presence of individuals with normal hearing with both types of exposure justify the importance of measures in health promotion, prevention, and hearing surveillance. The findings highlight the importance of valuing the patients’ own perception as the first indication of tinnitus and hearing loss in order to help develop appropriate public policies within the Unified National Health System (SUS).

## Introduction

Tinnitus is an endogenous, illusory sonorous sensation, result of an physiological alteration, that appears to be a sound, but is perceived in the absence of external sound stimuli. It is the first signal of a high sonorous stimulus. Tinnitus is commonly found along with hearing loss, either noise induced or not and can be classified according to its properties like its duration (seconds or minutes, intermittent or continuous), duration in time (days, months years) and graveness (annoyance level or life daily interference) [1]. Occasionally, everyone experience a transient sensation of tinnitus, which ceases spontaneously a few seconds later. However, when tinnitus becomes permanent, occurring frequently and having longer duration, may be a marker of quality of life worsening. It can be caused by numerous otological, metabolic, neurological, cardiovascular, pharmacological, dental, and psychological conditions, side effects of medications, and possibly the ingestion of drugs, caffeine, and alcohol [2], besides acting as a factor with major negative repercussions on the individual’s quality of life, interfering with sleep, concentration on daily and professional activities, and social life [2]. It affects approximately 17% of the world population, including 33% of the elderly [3-5]. Based on these data, approximately 7.6 million Brazilians complain of tinnitus, considered the third worst symptom affecting humans, exceeded only by intense and intractable pain and dizziness [6].

Tinnitus is the single or principal symptom involved in various diseases that can jeopardize health and well-being. Thus, special attention is needed in the case of normal hearing, since tinnitus can be the first symptom of diseases that are generally diagnosed by the presence of hearing loss [7].

According to the World Health Organization (WHO), “Health is a state of complete physical, mental, and social well-being, and not merely the absence of disease or infirmity.” Since the workplace is where workers spend major part of their time, maintaining health depends on a major extent of workplace healthiness. According to WHO, about 40% of European union (EU) population is exposed to environmental noise, 20% of these higher than 65 dBHL [8]. Unhealthy sound is defined under a specific national standard of the Brazilian Ministry of Labor, NR15, which characterizes occupational noise exposure, that is, worker is daily exposed to, at least, 85dBHL noise during his 8 hours labor day. Environmental noise exposure got its nomenclature after NBR 10152, which defines noise levels for acoustic comfort [9], more restrictive than occupational exposure ones. According to NBR10152, it’s defined by acoustic comfort level a 30-65dBHL maximum noise exposure.

In relation to occupational exposure, noise is the most common cause of tinnitus, and is also the main harmful physical agent in the workplace [10], as corroborated by the World Health Organization [11], which considers it an important problem for workers worldwide, due to its high prevalence.

Some authors evaluated noise exposure psychosocial effects on workers [10,12]. They found a connection between tinnitus and hearing loss, and a dose–response relation. Similar results were also found on Singapore [13] and England [14] workers with occupational noise exposure history. Among workers with a history of occupational noise exposure in Singapore [13], 23.3% reported tinnitus, as compared to 29.7% in a study in England [14]. A random sample study at Salvador city, Brazil [15], analyzed 720 individuals 15–90 years range and showed a 25% tinnitus rate, which differs somewhat from the literature (showing a prevalence of 15% in the general population and 33% in the elderly). From the workers who reported tinnitus, 86% also had hearing impairment.

The auditory effects (cochlear hearing loss, irreversible) and extra-auditory effects of noise exposure result in a clear decrease in quality of life [12,16], especially in workers who may experience difficulties in perceiving sound signals and locating sound sources from distance, in addition to hindering social relations due to difficulty caused by the hearing loss [12].

Noise-induced hearing loss (NIHL) is considered one of the ten principal etiologies of hearing loss in population, and among all auditory injury causes, it is the one with the best possibilities for prevention [16,17]. In addition, 85 to 96% of individuals with tinnitus show some concurrent degree of hearing loss, and both can have important repercussions on daily life [18]. Another major aspect of human auditory function is the high percentage of workers complaints related to difficulties in oral communication, their capacity for speech recognition and intelligibility [19].

Considering the related above, and in search of a more precise evaluation of workers’ health, the need to develop appropriate indicators for hearing health, and the institutional calling of a public research center in occupational audiology, a study was conceived, based on workers’ self-reported tinnitus, to identify the complaints and audiometric profile as well as relate them to the sound exposure types (environmental or occupational). This epidemiological survey results might contribute significantly to the audiology study field. Nowadays in Brazil, occupational exposure-related studies characterize hearing loss profile, hearing compromising level and their connection with hearing loss risk groups, specifically. In this study the group aim to correlate not only the hearing loss presence, but analyze the speech recognition index (SRI) in occupational and environmental exposed workers as well.

## Material and methods

This was an epidemiological cross-sectional survey, conducted from 2003 to 2007 at the Audiology Department of the Center for Studies on Workers’ Health and Human Ecology (CESTEH/ENSP/FIOCRUZ), a federal public institution.

### Sample

The potential study population consisted of 760 workers with hearing complaints who showed up spontaneously to evaluate their hearing condition. The final study sample included 495 workers (65.1% of the original total) that reported exposure to occupational or environmental noise plus tinnitus, all fit to undergo audiometric examination. The other 265 (34.9%) were excluded from the sample, since they did not report tinnitus complaints.

Classification of the origin of sound exposure, namely environmental [9] versus occupational [20], was based on the workers’ report of their job/workplace concerning the presence of a specific noise source during the labor day. Were not included cases with simultaneous exposure (occupational plus environmental).

### Occupational exposure selection criteria

According to NR 15 [20], workers undergoing unhealthy activities, which means, the ones daily exposed to high sonorous pressure (above 85 dBHL) for, at least, 8 hours per day in their workplaces, must be submitted to audiometric evaluation.

### Environmental exposure selection criteria

For environmental exposure classification, including work areas that are known to be noisy, like construction, transportation, industry, or laboratories, it was observed that workers related it as lower noisy exposure.

### Procedures

The workers underwent tonal and vocal audiometry, preceded by a clinical and occupational history (prepared by the department), inspection of the ear canal, and 14 hours of acoustic rest [21]. We sought to identify associations between the principal complaints, for which the following items from the case history were selected and analyzed statistically: socio-demographic data, hearing history, sonorous perception and perception difficulties, speech perception, and auditory signs and symptoms.

The tone thresholds were studied for air conduction at frequencies of 250 to 8000Hz and for bone conduction at frequencies of 500 to 4000Hz. The resulting hearing profile was classified as normal hearing or hearing loss: hearing thresholds were considered normal up to 25 dB HL [19,22], and logoaudiometric thresholds (SRI) were studied without the presence of competing noise to evaluate speech intelligibility [12]. This methodology aimed to select hearing status, distinguishing between normal hearing and hearing loss, so it did not involve the exclusive selection of profiles consistent with noise-induced hearing loss. Independent of the sample age, the qualitative and quantitative SRI analysis takes into account the 500, 1000 and 2000Hz range frequency specter, therefore there are no interferences over this article’s discussed data [19].

### Instruments

Both instruments and audiometer were calibrated and checked according to the national [21] and international (ISO) [23] standards. The audiometric tests were performed using a Beltone 2000 audiometer (ANSI-69 standard) and TDH 39 earphones, all submitted to annual calibration according to the ISO R 389 standard (1998) for air conduction and ISO 7566 (1987) for bone conduction.

### Questionnaire

The questionnaire was developed and adapted [24] by the speech therapists team at the Occupational Audiology Department, CESTEH/ENSP/FIOCRUZ, Rio de Janeiro, and has been applied since 2002 in over 2,000 patients. This questionnaire, which focuses on: socio-demographic data, personal habits, work market situation, social security benefits, environmental exposure, occupational exposure to noise and chemical substances, auditory identification (analysis of hearing acuity, onset of symptoms, difficulties on locating and noticing sound sources direction and speech perception), psychosocial evaluation, hearing history, hearing-related symptoms, non-auditory events, history of illness, and bother related by the worker when given the alternatives to define it according to the tinnitus duration, classifying it in mild, moderate or intense, in silence and after the workday. The three different tinnitus intensity levels identification was adapted based on Burden of disease from environmental noise, from WHO, which classifies tinnitus according to its attributes [1].

The following variables from the original questionnaire were analyzed: gender, work area (administrative, services, construction, domestic, industry, laboratory, health, transportation, and others), signs and symptoms like: difficulties on locating and noticing sound sources direction (bells and telephones), speech perception (closer, stronger, louder, in front, need for repetition, in a silent environment, background noise in front of radio or television, and when answering the telephone), tinnitus, and other non-auditory signs and symptoms like dizziness, vertigo, fainting, nausea, vomiting, and sweating, where the worker is allowed to report more than one symptom.

On the “workplace” variable the last activity related by the worker was considered, however all workers were exposed to occupational noise at any moment of their work lives.

### Statistical analysis

The variables gender and work area were submitted to descriptive analysis according to occupational versus environmental exposure (frequency). Descriptive analysis (means and standard deviations) were used for the variable “speech recognition index” in the right and left ears, according to the hearing profile (normal hearing and hearing loss). Data were submitted to two-factor ANOVA and Tukey statistical tests. Tinnitus was the independent variable; “occupational and environmental exposure” and “hearing loss and normal hearing” were the dependent ones. All the analyses set statistical significance at α=5%.

The resulting data were keyed in, consolidated, and analyzed using EPI-AUDIO software, developed by the Occupational Audiology Department team, CESTEH/ENSP/FIOCRUZ, Rio de Janeiro [25] based on Epi Info TM ®, made available by the CDC (Centers for Disease Control and Prevention) [26].

The study was approved by the Institutional Review Board of FIOCRUZ, case number 107/5, and included the guidelines of Brazilian Ruling 196/96 on research involving human subjects.

### Findings

#### Description of the study population

From 760 workers, 495 (65.1%) reported perceiving some intensity of tinnitus, of whom 50.4% classified it as mild, 23% moderate, 22.4% intense, 2.0% in the presence of silence, 0.4% after the workday, and 1.8% unspecified. Of these workers, 365 (73.7%) reported occupational exposure, of whom 261 (71.5%) were males, with a mean age of 46 years (SD = 12.34) and mean time on the job 15 years (SD = 12.76). Environmental exposure included 130 workers (26.3%), of whom 85 (65.4%) were females, with a mean age of 47 years (SD = 15.78).

It was thus observed a predominance of females (65.4%) in environmental exposure and males (71.5%) in occupational exposure. Concerning the workplace, approximately 30% of the workers with environmental exposure did domestic work, while for occupational exposure, 30% of the affected workers worked in construction jobs (Table 1). Both expositions had a, at least, 8 hours labor day.

**Table 1:** Distribution of socio-demographic characteristics of work

**Variable**	**Categories**	**Environmental exposure**		**Occupational exposure**	
		**n**	**%**	**n**	**%**
**Gender**	Female	85	65.4	104	28.5
	Male	45	34.6	261	71.5
**Work area**	Administrative	23	17.7	38	10.4
	Services	7	5.4	12	3.3
	Construction	6	4.6	110	30.1
	Domestic	39	30.0	47	12.9
	Industry	1	0.8	60	16.4
	Laboratory	18	13.8	10	2.7
	Health	3	2.3	9	2.5
	Transportation	2	1.5	29	7.9
	Other	31	23.8	50	13.7
**Total**		130	100.0	365	100.00

### Hearing profile analysis

According to Table 2, when we analyzed SRI for the groups of workers (hearing loss and normal hearing) and exposure (occupational and environmental noise), all the associations proved statistically significant. Workers with hearing loss showed a mean SRI of approximately 85%, as compared to mean SRI greater than 93.5% in workers with normal hearing, indicating that, irrespective of occupational exposure, there is speech perception impairment when the individual have hearing loss. When analyzing SRI according to the age groups a SRI reduction is found as the age progresses for the right (F = 12,36; p < 0,01) and left (F = 7,45;p < 0,01) ears.

**Table 2:** Mean SRI for each ear in workers with occupational and environmental noise exposure according to audiometry

**Environmental noise**	**Hearing Loss**					
	**(n 90)**					
	**Age groups**					
	**20-41**		**42-51**		**52-85**	
**Mean (S.D.)**	SRI RE	SRI LE	SRI RE	SRI LE	SRI RE	SRI LE
	87,64	89,16	81,96	85,92	83,69	85,79
	(18,68)	(16,08)	(19,70)	(19,81)	(16,86)	(14,76)
	**Normal Hearing**					
	**(n 40)**					
	**Age groups**					
	**20-41**		**42-51**		**52-85**	
**Mean (S.D.)**	SRI RE	SRI LE	SRI RE	SRI LE	SRI RE	SRI LE
	98,48	97,52	97,78	97,33	97,60	96,40
	(2,96)	(4,29)	(2,91)	(6,63)	(3,86)	(5,15)
**Occupational noise**	**Hearing Loss**					
	**(n 269)**					
	**Age groups**					
	**20-41**		**42-51**		**52-85**	
**Mean (S.D.)**	SRI RE	SRI LE	SRI RE	SRI LE	SRI RE	SRI LE
	89,33	89,85	86,59	85,07	80,03	83,41
	(15,63)	(12,08)	(15,44)	(16,42)	(17,21)	(15,43)
	**Normal Hearing**					
	**(n 69)**					
	**Age groups**					
	**20-41**		**42-51**		**52-85**	
**Mean (S.D.)**	SRI RE	SRI LE	SRI RE	SRI LE	SRI RE	SRI LE
	92,02	92,93	96,00	96,84	96,00	96,00
	(20,38)	(20,75)	(3,77)	(4,13)	(4,62)	(5,66)

Environmental noise: Hearing loss RE x Normal hearing RE (F = 23.16; p < 0.01).

Hearing loss LE x Normal hearing LE (F = 15.24; p < 0.01).

Occupational noise: Hearing loss RE x Normal hearing RE (F = 13.95; p < 0.01).

Hearing loss LE x Normal hearing LE (F = 16.56; p < 0.01).

As shown in Table 3, the associations’ investigation shows that among normal hearing workers who reported occupational noise exposure and tinnitus, 83,1% reported difficulties in sound localization, speech perception, and the presence of one or more non-auditory symptoms. Non-auditory symptoms were considered: tachycardia, insomnia, anxiety, irritation and difficulties in concentration and attention. Among hearing loss workers with tinnitus and occupational noise exposure symptoms, 79,7% reported difficulties in sound localization, 51,5% in speech perception and non-auditory signs and symptoms. When non-auditory signs and symptoms, speech perception and interference in sound localization were compared to the type of noise exposure (environmental versus occupational), there were no statistically significant associations.

**Table 3:** Reported signs and symptoms according to environmental and occupational noise exposure, for normal hearing and hearing loss individuals

	**Normal hearing**				**Hearing loss**			
	**Occupational**		**Environmental**		**Occupational**		**Environmental**	
	**n**	**%**	**n**	**%**	**n**	**%**	**n**	**%**
Difficulties on locating and noticing sound sources direction	250	84.50%	46	15.50%	55	79.70%	14	20.30%
Speech perception	242	81.80%	54	18.20%	35	50.70%	34	49.30%
Non-auditory symptoms	246	83.10%	50	16.90%	36	52.20%	33	47.80%

*Χ*^2^ = 0.45; p = 0.05.

## Discussion

The instrument used for data collection and analysis aimed to identify age range, gender and labor time, relate occupational/environmental sonorous exposure existence, in addition to characterizing difficulties in sound localization, speech perception, and the principal non-auditory signs and symptoms in workers with tinnitus as their principal complaint.

Other studies have been performed on similar populations [10,12]. The first article, on the psychosocial effects of noise, evaluated 32 male workers exposed to noise, with 20 years mean labor time. Of these, 62.5% had acquired neurosensory hearing loss, and the principal complaint was tinnitus (68.7%). The second author evaluated 284 workers also exposed to noise, without distinguishing industrial or non-industrial exposure, and found prevalence rates of approximately 48% for tinnitus and 63% occupational exposure. The author concluded that there is not only an association between hearing loss and tinnitus, but also a dose–response relationship. The study sample was 70.7% male and 29.2% female, with 15.6 years mean labor time and mean age 42.5 years.

Authors [14] have reported a tinnitus prevalence rate of some 20% in workers with a history of occupational noise exposure. In our sample, however, the prevalence rate was 65.1%, far higher than reported elsewhere in the literature. This high percentage is probably related to the fact that the study focused on workers with hearing complaints and the demand reported by workers during the case work-up, the symptoms’ subjectivity, and the fact that no test was performed to assess the possibility of other influences (since it was not part of the objectives in the study design). This was also an experienced sample, with a mean age of 42.5 years and mean labor time of 15 years, despite the type of noise exposure.

In a Master’s thesis on the influence of noise spectrum on the prevalence of noise-induced hearing loss and tinnitus [5], 192 hearing tests were performed, preceded by an occupational case history. The author found a 45.8% rate of reported tinnitus, and the highest prevalence of tinnitus (56.4%) occurred in workers (49.0% of the sample) that presented audiometric results consistent with noise-induced hearing loss.

The symptom is the factor that leads to the most difficulties and scarcity of data in studies on tinnitus, along with some other problems such as: the fact that tinnitus is a symptom rather than a disease; the lack of objective measurement methods; lack of adequate experimental models; and variations in the individual’s emotional or physical status [10]. The evaluation that can be used to record tinnitus is acuphenometry [27], but there is still no specific test for diagnosing tinnitus [14]. However, speech therapy clinical practice relies on the patient’s perception of tinnitus, which is subjective and displays wide individual variability in self-reporting. This highlights the importance of the case history and physical and audiometric examination.

In our study, the analysis of hearing quality in workers with tinnitus showed that in occupational noise exposure, the prevalence of hearing loss was higher (79.6%) than that of normal hearing (20.4%). The same was true for tinnitus and environmental noise exposure, that is, a higher prevalence of hearing loss (69.2%) as compared to normal hearing (30.8%).

Similar results for hearing loss were detected in a retrospective study of 358 patients examined from January 1995 to June 1999 by the research group on tinnitus at the University of São Paulo (HCFMUSP) [28] in which clinical hearing loss was reported by 60.4% of patients.

A study on individuals with tinnitus and normal hearing [7] showed this is an important group to investigate, since the findings are not influenced by hearing loss. There are few studies in this field, and there are no longitudinal studies on the evolution of these patients. In a search for answers to these questions, 36 patients were selected from the same research group as in the previous study, from 1995 to 2003, who presented normal audiometry when they were included for follow-up. Although the study showed neither worsening of tinnitus over time nor significant changes in its characteristics, an important share of the sample evolved to hearing loss (7.4%).

In our study on workers with tinnitus, for both types of noise exposure (environmental and occupational), the majority of patients presented hearing loss; however, the proportion of patients with normal hearing was higher in cases of occupational noise exposure. This result is especially important when considering the findings that indicate evolution to hearing loss [7] and studies by other authors [5,16] highlighting tinnitus as the first symptom of hearing dysfunction.

A literature review shows numerous articles in the occupational field describing the prevalence of hearing loss, for example a study in Greater Metropolitan Salvador, Bahia State, Brazil [29] in 7,925 workers from 44 factories in nine different branches of industry, showing 45.9% prevalence of hearing loss.

In marble workshops in Brasília, Federal District of Brazil, the prevalence of hearing injury was 48.0% [30]. In the city of Goiania, a study in a metallurgical factory with 187 workers found a 22% prevalence of hearing loss, suggestive of noise exposure [31].

In São Paulo State, noise control programs in four metallurgical factories in the city of Piracicaba [32], with a total of 741 workers, were analyzed, showing 41% prevalence of hearing alterations, with workers’ mean age 42.3 years and 16.7 years mean labor time.

Another highly relevant question is the measurement of hearing function in relation to speech intelligibility in the audiological battery normally used in audiometric tests. In most routine examinations the battery is considered incomplete, since it does not include measures of speech recognition [19]. Thus, in the attempt to understand these and other aspects, studies have been performed with the presence of competing noise and other factors.

One example [33] is a prospective clinical study in 60 adults, divided into 3 groups (20 with normal hearing, mean age 23.3 years; 20 with hearing loss, mean age 40.4 years; 20 elderly with hearing loss and mean age 66.8 years), aimed at investigating the effects of hearing loss and age on speech recognition in the presence of ipsilateral competing noise. The study showed a mean SRI proportion of 92% for all the groups studied, that is, no major difference was found between the three groups in terms of intelligibility performance, since the hearing thresholds at the speech frequencies were preserved. In our study, the mean SRI values in both the right and left ears were consistent with both the literature [19,34] and the observed hearing conditions, not concerning the type of exposure. The values varied from 84.2% to 86.7% for hearing loss and from 93.5% to 98.1% for normal hearing, thus representing a limited number of failures in speech intelligibility. Even with values close to 88%, some speech recognition impairment is expected [19].

The observed hearing loss values reflect a small percentage-wise alteration in speech intelligibility, consistent with the literature [19,34] and with the above-mentioned study [33], although the latter was not applied to individuals with tinnitus.

These minor alterations in speech recognition contrast with the high rates of difficulty in speech perception identified in normal hearing individuals complaining of tinnitus with occupational noise exposure (81.8%).

In 1986, Tyler & Baker [35] described the interrelationship between tinnitus and daily activities. In a public referral service for tinnitus, 49.3% of the 358 patients examined [28] reported impaired concentration in activities of daily living and 14.2% in social activities. In a previous study [36], the same service conducted a retrospective study of 150 patients in the Tinnitus Outpatient Clinic of the Department of Clinical Otorhinolaryngology at the FMUSP University Hospital and found that 76% reported some alteration in at least one activity of daily living, and that 47.3% reported difficulties in concentration. Our study found prevalence rates of 84.5% for sound localization difficulty (doorbell and telephone), 81.8% for speech perception (silent environment/presence of background noise), and 83.1% for otological signs and symptoms. These percentages in individuals with tinnitus with occupational noise exposure were similar to those found in environmental exposure. It is important to note that studies cited by Sanchez et al. [36] refer to a tinnitus specialist public ambulatory.

### Study’s constraints

Since it is a cross-sectional study, the chronological relation between the events may not be easily detectable. It is about occupational/environmental noise exposed workers, having specific complaints like tinnitus, welcomed at a specific workers’ health Center. Age range shall not be considered as bias for hearing loss and tinnitus, since the age group includes hearing loss workers with ages below 30 years. Moreover, SRI evaluation methodology used corroborates for the findings.

As information bias, external factors as earplug use and vehicle, home and free-time noise may not be related.

## Conclusion

In this study, when we analyzed the mean speech recognition index (SRI) for the groups of workers (with hearing loss versus normal hearing), age groups and types of exposure (occupational versus environmental noise), we found lower SRIs for workers with hearing loss, thus characterizing greater difficulty in speech recognition for cases of occupational hearing loss.

Another finding was the presence of individuals with normal hearing in both types of noise exposure, which highlights the importance of health promotion, prevention, and hearing surveillance measures, especially considering the fact that tinnitus can be the first symptom of diseases that are usually diagnosed on the basis of hearing loss. In relation to environmental exposure, this finding is especially important due to lack of recognition of environmental noise risks for workers. There is no legal control of workers’ exposure to environmental noise, which emphasizes the need for awareness-raising campaigns in defense of environmental sound quality, regardless of the origin of the noise, as well as the need for surveillance measures to monitor hearing in the exposed population. The existing Brazilian legislation [20] only deals with the built-up environment.

The findings highlight the importance of valuing the workers’ own reported complaints in developing public policies (or educational programs) for the early detection of tinnitus and hearing loss, given that self-perception can be the first indication of tinnitus and hearing loss. Workers’ self-report is an inexpensive and preventive technique, which can minimize the costs for the Unified National Health System (SUS) in workers’ treatment and rehabilitation.

## Competing interests

The authors declare they have no competing interests.

## Authors’ contributions

MS participated in the study conception and writing of the article. LFR worked on the data collection in the occupational case histories and on the hearing evaluation. DFRV worked on keying in the data in the database and the data analysis and interpretation. LRT and DV were responsible for the statistical processing and revision of the article. VF and DV were responsible for the overall and final revision of the article. All of the authors participated in the various stages in the elaboration of the text.
